# Wnt Signaling in Renal Cell Carcinoma

**DOI:** 10.3390/cancers8060057

**Published:** 2016-06-17

**Authors:** Qi Xu, Mirja Krause, Anatoly Samoylenko, Seppo Vainio

**Affiliations:** Biocenter Oulu, Faculty of Biochemistry and Molecular Medicine, Oulu Center for Cell Matrix Research, University of Oulu, P.O.Box 5000, FI-90014 Oulu, Finland; xu.qi@oulu.fi (Q.X.); mirja.krause@oulu.fi (M.K.); Anatoliy.Samoylenko@oulu.fi (A.S.)

**Keywords:** renal cell carcinoma, Wnt signaling pathway, therapeutic target, development

## Abstract

Renal cell carcinoma (RCC) accounts for 90% of all kidney cancers. Due to poor diagnosis, high resistance to the systemic therapies and the fact that most RCC cases occur sporadically, current research switched its focus on studying the molecular mechanisms underlying RCC. The aim is the discovery of new effective and less toxic anti-cancer drugs and novel diagnostic markers. Besides the PI3K/Akt/mTOR, HGF/Met and VHL/hypoxia cellular signaling pathways, the involvement of the Wnt/β-catenin pathway in RCC is commonly studied. Wnt signaling and its targeted genes are known to actively participate in different biological processes during embryonic development and renal cancer. Recently, studies have shown that targeting this pathway by alternating/inhibiting its intracellular signal transduction can reduce cancer cells viability and inhibit their growth. The targets and drugs identified show promising potential to serve as novel RCC therapeutics and prognostic markers. This review aims to summarize the current status quo regarding recent research on RCC focusing on the involvement of the Wnt/β-catenin pathway and how its understanding could facilitate the identification of potential therapeutic targets, new drugs and diagnostic biomarkers.

## 1. Introduction

Kidney cancer is the ninth most common cancer in men worldwide, and renal cell carcinoma (RCC) is thought to be the main kidney malignancy with higher occurrence in males than females [1]. The occurrence rate varies between countries and still has been increasing in most regions, like Central and South America, during the past 10 years [1]. RCC is revealed as a heterogeneous group of tumors, which are classified into subtypes of which clear cell RCC (ccRCC) (75%–80%) is the most common one. Other prominent forms are papillary (10%–15%), chromophobe (5%) and collecting duct (1%) RCC [2]. The poor diagnosis of early stage kidney cancer and resistance to both traditional chemotherapy and radiation therapy are the cause of treatment failure in patients with renal cancer. Therefore, understanding the molecular mechanisms during the initiation and the development of RCC is of great importance, and findings could be used to help identify therapy strategies in kidney cancer. PI3K/Akt/mTOR, HGF/Met, VHL/HIF and Wnt signaling pathway members are known to be involved in RCC. Ectopic regulation of the Wnt signaling pathway by DNA methylation [3] or mutation [4,5,6,7] can induce changes in the expression of the Wnt signaling molecules, which are linked to renal malignancy. These findings highlight the potential prognostic and therapeutic value of especially the Wnt signaling pathway in RCC. In this review, we summarize the regulatory mechanisms of the Wnt signaling pathway and its downstream effectors in RCC. Finally, we aim to emphasize the importance of Wnt signaling as a possible drug target, which may facilitate better treatment of RCC and the development of improved therapies.

## 2. Wnt Signaling Pathway

**Figure 1:**
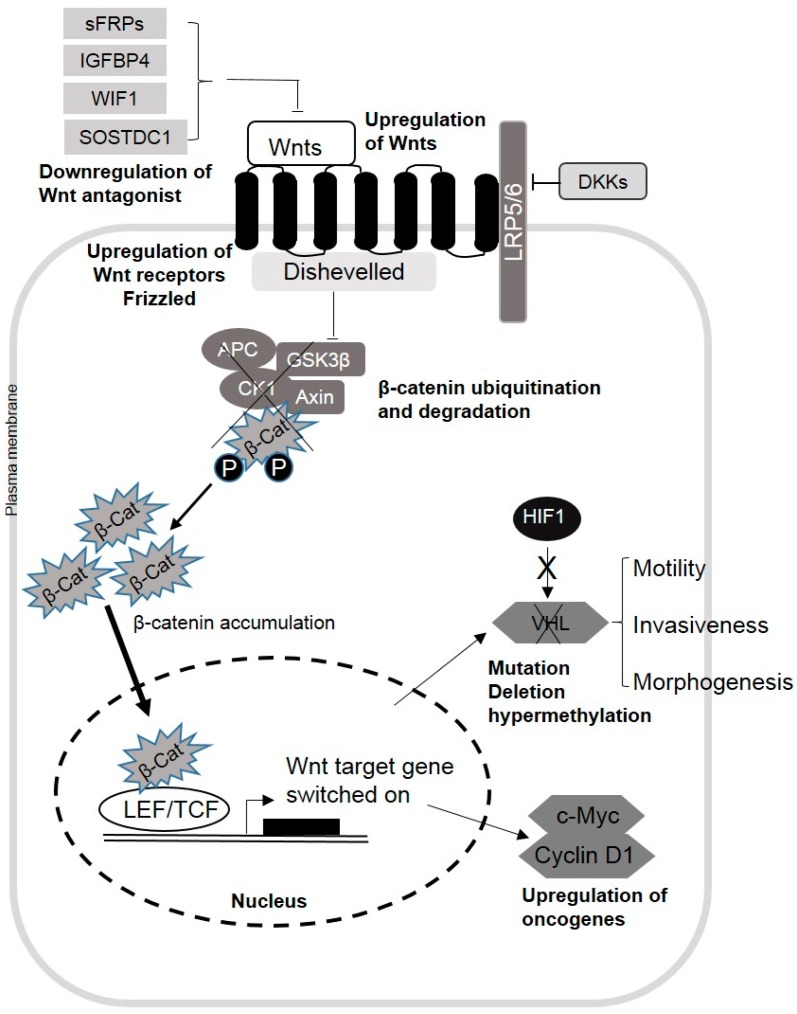
Schematic representation of the Wnt/β-catenin signaling pathway in renal cell carcinoma (RCC). Wnts binding to its receptors from the Frizzled family stimulates the canonical signaling pathway. The downregulation of Wnts and its co-receptor LRP5/6 antagonists by gene mutation, deletion and promotor hypermethylation also induces the Wnt/β-catenin signaling pathway in RCC. Upon the activation of the pathway, GSK3 kinase activity is inhibited, and the destruction complex becomes disrupted. This allows β-catenin accumulation in the cytoplasm and its localization to the nucleus, which activates Wnts target genes’ transcription. Upregulation of the Wnt/β-catenin signaling pathway increased the expression of the oncogenes in RCC, such as c-Myc and Cyclin D1. The VHL/HIF pathway is regulated by the Wnt/β-catenin signaling pathway in RCC. The tumor suppressor gene VHL can be silenced by mutation, deletion and promotor hypermethylation. LRP5/6: LDL receptor-related proteins 5 and 6; GSK3: glycogen synthase kinase 3; TCF/LEF: T-cell factor/lymphoid enhancing factor; VHL: von Hippel-Lindau.

The Wnt family includes 19 secreted glycoproteins, which regulate cell proliferation, differentiation, survival, migration and stem cell self-renewal [8,9,10]. In development biology, the Wnt signaling pathway has been extensively studied during the past decades and can mediate biological processes either by the canonical or the noncanonical pathway, depending on the involvement of β-catenin in the signal transduction. β-catenin, an intracellular signal transducer, is a core component in the cadherin protein complex and its stabilization is essential for the activation of the Wnt/β-catenin signaling pathway (Figure 1) [11]. In the activated canonical Wnt signaling pathway, Wnt proteins are secreted and bind to its appropriate membrane receptors from the Frizzled (Fzd) family together with the co-receptors LDL receptor-related proteins 5 and 6 (LRP5/LRP6), which are required for the recruiting of cytoplasmic phosphoprotein Disheveled (Dsh/Dvl) [12]. The formed Wnt/receptor complex will disrupt the “destruction complex” that contains adenomatous polyposis coli (APC), AXIN and glycogen synthase kinase 3β (GSK-3β) by recruiting AXIN [13]. The interaction of Wnt and its receptor leads to the stabilization and accumulation of β-catenin in the cytoplasm, as well as its translocation into the nucleus, inducing the downstream gene expression together with the cofactors T-cell factor/lymphoid enhancing factor (TCF/LEF) [14]. The “destruction complex” is formed in the absence of Wnt proteins, leading to the degradation of β-catenin molecules by ubiquitination and proteasome digestion. The Wnt/Ca^2+^ pathway and the Wnt/planar cell polarity (PCP) pathway are two noncanonical Wnt signaling pathways [15]. Both pathways require the binding of Wnt proteins to the receptor Fzds before the signal is transduced to cytoplasmic phosphoprotein Dsh/Dvl, but without the use of LRP5/6 as its co-receptor [16]. The PCP pathway activates c-Jun N-terminal kinase (JNK) and the Ras homolog gene family member A (RhoA) cascade, which targets the genes that control rearrangements in the cytoskeleton [15]. Unlike the PCP pathway, heterotrimeric G proteins and phospholipase C (PLC) are activated by the Wnt-Fzd complex in the Wnt/Ca^2+^ pathway. The increased intracellular Ca^2+^ concentration and activation of effectors regulate downstream gene transcription in controlling cell fate, cell adhesion and cell migration.

The Wnt signaling pathway plays an important role both in embryonic development and carcinogenesis. Its role during embryonic development has been reported in the kidney [17,18,19,20], lung [21], genital system [22,23,24,25], teeth [26,27,28,29,30], pancreas [31,32], brain [33], mammary gland [34], skin and neuromuscular junctions [35,36]. The signaling pathway regulates developmental processes, such as cell proliferation, apoptosis, migration and stem cell self-renewal/differentiation. Dysregulation of the signaling pathway and its effectors causes different degenerative diseases, as well as cancer [37,38]. The Wnt signaling pathway plays a crucial role in regulating kidney organogenesis by controlling both ureteric bud (UB) development and serving as an inductive factor to regulate nephrogenesis in mesenchymal cells. Conventional knock-out studies, like Wnt4, Wnt5a and Wnt11 deletion in an embryo, cause severe kidney phenotypes and lethality [39,40,41].

It has been shown that the dysregulation of Wnt signaling contributes to the development of human cancers, including colorectal, ovarian, breast cancer and RCC [6,42,43]. Various cellular functions, such as apoptosis, proliferation, migration and invasion, are involved in Wnt-dependent carcinogenesis processes [7]. The Wnt signaling pathway was reported in epithelial-mesenchymal transition (EMT) in embryonic development and carcinogenesis, which facilitate the cell migration and formation of metastases. It is also believed that the properties of cancer stem cells are regulated by the evolutionarily-conserved signaling pathways common also in somatic stem cells, including Wnt/β-catenin [44].

## 3. The Wnt Signaling Pathway in Renal Cell Carcinoma

The important role of Wnt signaling in RCC is highlighted by the fact that the expression of different Wnts, Wnt receptors (Fzds) and Wnt antagonists is altered in human RCC (Table 1). In ccRCCs, high Wnt1 expression was associated with increased tumor diameter, more advanced stage and invasiveness [45]. Wnt10A expression was also significantly increased in RCC cell lines and tissues being independent risk factors for renal carcinogenesis [46]. On the other hand, the downregulation of Wnt7A gene expression was detected in the majority of ccRCCs, while methylation analysis revealed positive correlations between tumor stage and Wnt7A hypermethylation [47]. Low levels of Wnt5A can be also associated with kidney tumor development [48]. These differences in expression probably reflect the variability of downstream signaling mechanisms in the Wnt family. While Wnt1 and Wnt10a are canonical Wnts, Wnt5a and Wnt7a belong to the non-canonical group [49,50]. The differences between these two groups of Wnts were found also for other tumor types. For instance, Wnt-1, but not Wnt-5A and Wnt-7A, activated the TCF reporter gene and β-catenin stabilization in esophageal cancer cells [51]. In ameloblastomas, most canonical Wnts were found to be overexpressed, while non-canonical and indeterminate Wnts were mostly absent [52].

The mRNA levels of Wnt receptors Fzd5 and Fzd8 were increased in RCC when compared to the normal kidney tissue, which was followed by the increase of their downstream target cyclin D1, suggesting that Fzd5 and Fzd8 may have a role as biomarkers in RCC [53]. Moreover, the repression of Fzd5 by restoring miR-124 function overcomes P-glycoprotein-mediated chemoresistance in RCC [54]. Immunohistochemical analysis showed that the Fzd7 protein expression level was significantly increased in RCC when compared to the surrounding normal tissues, though this expression was not correlated with clinicopathological parameters [55].

Altered expression of β-catenin was also detected in RCC [56,57]. Cytoplasmic β-catenin was identified as the most promising candidate associated with unfavorable clinicopathology and impaired survival in RCC patients [45]. The patients with high cytoplasmic β-catenin levels were characterized by higher tumor diameter, more advanced stage and vascular invasion. However, it failed to reveal any genetic alterations in the β-catenin gene [56,57]. Another study confirmed that β-catenin mutations in RCC carcinoma are relatively rare. This study also argued that cytoplasmic accumulation of β-catenin protein is found only in about a quarter of ccRCC [58]. Still, a recent multilayer-omics analysis of RCC confirmed the important role of the Wnt/β-catenin signaling pathway in renal carcinogenesis [59]. Indeed, 36 genes that showed genetic aberrations, DNA methylation alterations and/or mRNA expression alterations in one or more RCCs were correlated with Wnt/β-catenin signaling in this study. While cytoplasmic β-catenin expression is frequently connected with unfavorable RCC development, nuclear expression does not seem to be associated with any clinicopathology [45]. These data may indicate that though transient nuclear translocation of β-catenin is necessary for the activation of Wnt-dependent gene expression, constant nuclear localization of β-catenin does not necessarily lead to higher activation of Wnt signaling. High cytoplasmic accumulation of β-catenin may be a sign of its abnormal stabilization.

Molecular mechanisms leading to the induction of cancer growth upon Wnt signaling dysregulation are not completely characterized yet (Figure 1). One of the common pathways that are induced upon Wnt1 signaling is the inhibition of cancer therapy-mediated apoptosis via β-catenin/TCF [60]. In accordance with this observation, it was found the impaired Wnt signaling together with changes in the TCF4 splicing isoform profile are associated with RCC progression by the inhibition of the apoptotic pathway [61]. Another study showed that Wnt10A gain-of-function by plasmid transfection induced RCC cell transformation, proliferation, migration, invasiveness and chemoresistance due to the activation of β-catenin-dependent signaling. Conversely, knockdown of Wnt10A expression by corresponding siRNA decreased the cell proliferation and aggressiveness of RCC cells [46].

Additionally, the aberrant Wnts signaling in RCC was also caused by the loss of function of Wnt antagonists, for example by the downregulation of WIF1 (Wnt inhibitory factor 1), members of sFRP (secreted frizzled-related protein) family, Dkk (Dickkopf) family, IGFBP4 (insulin-like growth factor-binding protein 4) and SOSTDC1 (sclerostin domain-containing protein 1) [62,63,64,65]. Wnt antagonists are vital in RCC pathogenesis, and their loss-of-function in RCC, for example by promotor hypermethylation, could lead to the constitutive activation of Wnt signaling, resulting in higher cell proliferation and differentiation during carcinogenesis [62]. Immunohistochemistry and qPCR revealed that WIF1 was significantly downregulated in RCC samples and RCC cell lines [66]. Similarly, genomic profiling, qPCR and immunohistochemistry indicated that loss of sFRP1 expression probably through methylation of this gene occurred in ccRCC and papillary RCC patients [67]. Stable re-expression of sFRP1 in cRCC cells resulted in slower cell growth, inhibition of anchorage-independent growth and decreased tumor formation in athymic nude mice. sFRP2, as a tumor suppressor, was shown to be inactivated by DNA methylation in RCC [68,69]. Another study similarly indicates that re-activation of sFRP2 with DAC-mediated inhibition of DNA methylation in RCC cells induces cell apoptosis [70]. These data draw attention to the fact that epigenetic alteration of sFRP2 in RCC changes the properties of RCC cells, and restoration of its expression by inhibition of DNA methylation could be a strategy for renal cancer therapy. On the other hand, a number of studies showed that sFRP2 promotes tumor progression. In particular, Yamamura *et al.* showed that overexpression of sFRP2 in A498 renal cancer cell lines activated the canonical Wnt pathway and promotes renal cell proliferation *in vitro* and tumor growth *in vivo* [71]. The discrepancy of these results with the ones described above might be connected with the fact that a number of genes that are not direct targets of the Wnt signaling pathway were found to be upregulated by sFRP2 overexpression. The authors suggested that ectopic sFRP2 expression can induce alternative signaling pathways in A498 cells, including the suppression of p53 signaling [71]. Among the pro-oncogenic effects that can be induced by sFRPs are the increased activity of metalloproteases [72]. In general, the intracellular concentration of SFRP1 and 2 together with the local availability of Fz, Wnts and downstream signaling components would probably determine the progression of carcinogenesis in each particular case [72].

Guo *et al*. [73] examined the Dkk1 and Dkk3 levels both at protein and the mRNA levels in human ccRCC and reported that Dkk1 and Dkk3 was significantly lower in human ccRCC than in the healthy controls. SOSTDC1 levels were reduced in adult renal clear cell tumors and pediatric Wilms tumors [74]. The function of IGFBP4, that is believed to be a Wnt antagonist, in RCC seem to be quite complex [75]. Indeed, expression of IGFBP4 was significantly lower in primary RCC, but higher in metastatic RCC compared to normal human kidney tissues. Moreover, IGFBP4 expression activates cell growth, metastasis and Wnt/β-catenin signaling in RCC cells and may promote tumor growth in mice [75].

Activation of the Wnt signaling pathway alters the expression of oncogenes and tumor suppressor genes in RCC either directly or via interaction with other pathways. Among the Wnt-regulated genes upregulated in ccRCC tissues are oncogene c-Myc and cell cycle regulator cyclin D1 [53,67,76]. The loss of von Hippel-Lindau (VHL) allowed the robust cell motility, invasiveness and morphogenesis by HGF-driven oncogenic beta-catenin signaling in familial and most sporadic ccRCC [77]. VHL plays a crucial role in the regulation of hypoxia-inducible factor (HIF) stability by targeting it for polyubiquitylation and proteasome degradation [78]. Interestingly, in contrast to many other tumor types, HIF-1α and HIF-2α have opposing effects in ccRCC biology, with HIF-1α acting as a tumor suppressor and HIF-2α acting as an oncogene. Therefore, the overall effect of VHL inactivation will depend on the fine-tuning of the HIF response [79].

Unlike the Wnt/β-catenin signaling pathway, the role of non-canonical Wnt signaling in RCC is less investigated. RTK-like orphan receptor 2 (Ror2), a Wnt ligand receptor, the expression of which is normally restricted to embryogenesis, was overexpressed in ccRCC [80]. Downregulation of its expression in RCC by shRNA knockdown or mutation could reduce the tumor growth, cell migration and cell invasion stimulated by Wnt/Rho signaling [80,81]. A Wnt antagonist, DKK3, was inactivated in RCC, while its overexpression not only inhibited cell proliferation, but also increased cell apoptosis [82]. In this study, DKK3 induced RCC cell apoptosis via the Wnt/JNK pathway [82].

## 4. Potential Therapeutic Targets

Due to the constant increase of cancer rates worldwide [83], tremendous resources are being invested in searching for less toxic, more selective and more effective drugs to be applied in anti-cancer therapies. Interestingly, 96% of RCC cases are sporadic, and only 4% are hereditary [6], making it very difficult to predict. Conventional treatments are usually radiation therapy, local ablation and surgery to remove part of or the whole kidney (American Cancer Society, 2016). However, RCC is difficult to diagnose in the early stage, as the symptoms are often noticed late, and most patients show advanced cancer by the time it is finally discovered [44]. Furthermore, RCC is not very sensitive to radiation therapy. Therefore, targeted drugs are often the first choice of treatment. They usually block angiogenesis or growth-stimulating molecules (e.g., tyrosine kinases) in the cancer itself [84,85]. Prominent examples for these types of drugs are sorafenib and sunitinib [86]. However, prolonged use of sorafenib or sunitinib would lead to drug resistance in human RCC [87]. A new product, ovatodiolide, which could target the Wnt/β-catenin pathway in RCC, was shown to have the possibility to overcome the resistance of sorafenib or sunitinib *in vitro* [88]. Genistein, another widely-used drug that targeted the Wnt signaling, was reported to be antiproliferative and antiangiogenic in human RCC [89,90]. In 2007, by using the human papillary RCC cell line KCI-18, Hillman and his colleagues established a reliable and predictable metastatic RCC tumor model in nude mice, and they showed that the combination of genistein with radiation inhibited the growth and progression of established kidney tumors [91]. Others, like temsirolimus and everolimus, target the cell protein mTOR (mammalian target of rapamycin), thus inhibiting cell division and growth [86]. These drugs can have heavy side effect by boosting the immune system, like hair loss or sickness. Introduction of new sequencing technologies, known as next generation sequencing (NGS), have positively influenced these efforts. NGS enabled scientists to now take a closer look at changes on the molecular and genetic levels of cancerogenesis, which will be crucial for providing genetically-driven care in this era of precision medicine [92]. Research aims to better understand the involvement and intervention in the pathogenic mechanisms of certain signaling pathways. Changes in proto-oncogenes and tumor suppressor genes can lead to erroneous signal transmission hence resulting in increased cell proliferation rates, initiating tumor formation and cancerogenesis. It has been found that alterations in pathway structures of the different pathways present in the cell (HGF/c-Met, PIK3/AKT/mTOR, Wnt/β-catenin and MAPK) can result in renal cancerogenesis [4,93]. Some of these pathways are already the target for therapeutic strategies. For example, the inhibition of the HGF/c-MET pathway is targeted by inhibiting the autophosphorylation of c-MET and also by suppressing of the signaling cascade normally activated downstream of c-MET [94]. As discussed earlier, also the Wnt signaling pathway is associated with RCC, making it a potential target in RCC treatment. It has been found that the inhibition of Wnt signaling in RCC can inhibit cancer cell proliferation and survival [62]. The aberrations in Wnt signaling and β-catenin expression in RCC are closely associated with RCC development. Three Wnt inhibitors (ethacrynic acid, ciclopirox olamine and piroctone olamine), which selectively inhibit Wnt signaling, were tested in two studies with and without the addition of bifunctional peptides. They showed the ability to induce RCC cell apoptosis and, by targeting β-catenin expression, reduce cancer cell survival [95,96]. These findings could possibly lead to the discovery of targeted anti-cancer drugs affecting the Wnt-signaling pathway, resulting in new treatment possibilities for RCC.

As mentioned earlier, Wnt/β-catenin signaling plays an important role in many cancers (e.g., colon, liver and breast cancer). Cancer stem cells, a small subpopulation of self-renewing cells that have the potential to form new cancer colonies, were discovered in different malignancies, including renal carcinoma [97]. In several types of malignancy, such as non-melanoma skin cancer and colon cancer, there are data indicating the contribution of Wnt signaling in the maintenance of the CSC population [98]. It was found that the three-dimensional culture of mouse renal carcinoma cells leads to an increase in cancer stem cell number and simultaneously to the upregulation of stem cell-like genes, such as those associated with the Wnt pathway [99]. Since Wnt signaling, together with several other pathways involved in embryonic development, has an important role in CSCs and it is believed that selective targeting of CSCs may achieve better antitumor effects as compared to conventional chemotherapy, significant efforts are made world-wide to develop potent Wnt signaling inhibitors.

The targeting of Wnt pathways for cancer therapies in general [100] and the identification of next biomarkers [101] have been recently reviewed. Among the inhibitors of the Wnt pathway that are developed for anti-cancer treatment are antibodies that neutralize Wnt ligands, those that inhibit the Wnt receptors, as well as agents targeting β-catenin [98]. At the moment, more than 10 different substances that target different components of the Wnt signaling pathway are in clinical development for the treatment of various cancers; some are already in phase II oncology trials [98]. These results make it probable that Wnt inhibitors can find also a role in the therapy of renal carcinomas. Blagodatski *et al.*, 2014 [100], also raises the important issue that only a few of the inhibitors identified have made it into clinical trials. This is due to the fact that a broad inhibition of the Wnt/β-catenin pathway is potentially risky, since Wnt pathways are not only involved in the maintenance of adult tissue homeostasis (e.g., the maintenance of the differentiated epithelium and its interaction with mesenchymal cells and the pluripotent state of stem cells), but also in developmental processes [100]. The probable side-effects from Wnt inhibition include negative effects on intestinal stem cells, bone turnover and hematopoiesis [102]. The question, if one can safely target the Wnt signaling pathway in cancer treatments, was recently addressed in detail in a review [102]. Therefore, it is important to remember that not just the search for an inhibitor is the task current research needs to address, but also a way to instead fine-tune/manipulate the dysregulated pathway back to its normal physiological state. Indeed, during normal physiological processes, the mechanisms of the downregulation of Wnt signaling by negative feedback loops are as important as the mechanisms of Wnt stimulation.

Developments in organ *in vitro* culture ease the studies on signaling in kidney tissue and during nephrogenesis [103]. An important method called reaggregation or 3D assay was established by Unbekandt *et al.* [104]. They isolated metanephric kidneys (metanephric mesenchyme (MM) and ureteric bud (UB)) between E11.5 and E13.5 and fully dissociated them by dissection and subsequent enzymatic treatment. Afterwards, they reaggregated the dissociated kidneys by centrifugation and were able to culture these aggregates using standard organ culture. The 3D assay can be also done with only dissociated-reaggregated MM, which is then induced by embryonic spinal cord [105]. In 2015, by inducing human iPS cells, Takasato and his colleagues successfully generated the kidney organoids in which the tubules, early loops of Henle and glomeruli vascularization could be observed [106]. These 3D kidney organoids can be easily manipulated (e.g., overexpression or downregulation of genes, *etc.*), being a simple way to study the task and roles of signaling molecules during kidney development. Therefore, the 3D assay is a promising tool in the search for new and different anti-cancer drug targets.

Very recently, it has been found that Wnt proteins can be associated with extracellular vesicles [107]. These membrane-enclosed vesicles are released by various different cell types and can be found in body fluids, such as blood and urine. A specific subgroup of vesicles is called exosomes (EXs). EXs are 30–100 nm and carry mRNA, miRNA, proteins and signaling molecules [108,109]. An increasing number of studies suggests that they are not only important in cell-to-cell communication, but are also involved in various physiological and disease processes. Recent findings show that EXs play a role in cancer-associated immune suppression and also immune response activation, in kidney regeneration and possibly kidney development, in renal tumor progression and metastasis, as well as cancer evolution (reviewed by [110]). Furthermore, it was found that EXs can induce the activation of the AKT and Erk1/2 pathways in the cell [111], and in breast cancer cells, EXs mobilize autocrine Wnt-planar cell polarity (Wnt PCP) signaling [112]. Many things are still unknown about the exact role of EXs and their association with Wnt proteins in particular. This, however, seems to be a promising area where new targets for anti-cancer drugs can be identified in the future. Moreover, this could lead to the discovery of new non-invasive diagnostic markers due to the fact that EXs are present in blood and urine and can easily be isolated. Additionally, EXs could be used as vaccines, which specifically target the tumor or the infected tissue, reducing the number of side-effects and the toxicity of current common treatments.

## 5. Conclusions

It still remains unclear how exactly and to what extent signaling pathways are involved in the formation and progression of RCC. Recent scientific advancement (e.g., NGS, 3D-assay) in addition to a refocus of current research towards a better understanding of the apparent dialogue between the molecules, which are part of the different cellular signaling pathways, holds great potential for the discovery of new therapeutic targets and diagnostic markers. It has become clear that Wnt signaling contributes to cancerogenesis and tumor progression. The communication between Wnt signaling and other pathways can be the reason that cancer cells are able to maintain growth and survival. Furthermore, a large amount of experimental evidence suggests that specifically targeting Wnt signaling to develop new therapies is a point to start. Here, the recent discovery that extracellular vesicles can intervene in the signaling cascades present in the cell, gives hope that maybe not only new drug targets can be identified, but non-invasive diagnostic tools enabling earlier discovery of RCC and novel treatment procedures, e.g., vaccines, can be developed in the near future.

## Figures and Tables

**Table 1:** Wnt signaling pathway components associated with human renal cell carcinoma.

Protein	Methods Used	Detection Level	Expression	Reference
**Wnt Family**				
Wnt1	IHC	Protein	High	[45]
Wnt5a	qRT-PCR	mRNA	Low	[48]
Wnt7a	Methylation-specific PCR, bisulfite DNA sequencing, qRT-PCR	mRNA	Low/hypermethylation	[47]
Wnt10a	IHC	Protein	High	[46]
**Wnt antagonist**				
sFRP1	ICH, qRT-PCR, bisulfite DNA sequencing, Western blot	Protein, mRNA	Low/hypermethylation	[69]
sFRP4/5	Methylation-specific PCR, microarray, qRT-PCR	mRNA	Low	[64,65]
Dkk1-3	ICH, qRT-PCR, Western blot	Protein, mRNA	Low	[65,82]
DKK4	qRT-PCR, Western blot	Protein, mRNA	High	[63]
IGFBP4	ICH, qRT-PCR	Protein, mRNA	Low	[75]
SOSTDC1	ICH, cDNA microarray	Protein, mRNA	Low	[74]
WIF1	ICH, qRT-PCR	Protein, mRNA	Low	[66,68]
**Wnt receptor**				
Fzd1	ICH, qRT-PCR	Protein, mRNA	Low	[67]
Fzd5/8	Western blot, qRT-PCR	Protein, mRNA	High	[53]
Fzd7	ICH	Protein	High	[55]

## References

[1] Znaor A., Lortet-Tieulent J., Laversanne M., Jemal A., Bray F. (2015). International variations and trends in renal cell carcinoma incidence and mortality. Eur. Urol..

[2] Zambrano N.R., Lubensky I.A., Merino M.J., Linehan W.M., Walther M.M. (1999). Histopathology and molecular genetics of renal tumors toward unification of a classification system. J. Urol..

[3] Shenoy N., Vallumsetla N., Zou Y., Galeas J.N., Shrivastava M., Hu C., Susztak K., Verma A. (2015). Role of DNA methylation in renal cell carcinoma. J. Hematol. Oncol..

[4] Banumathy G., Cairns P. (2010). Signaling pathways in renal cell carcinoma. Cancer Biol. Ther..

[5] Clevers H. (2006). Wnt/Beta-catenin signaling in development and disease. Cell.

[6] Cojocaru E., Lozneanu L., Giusca S.E., Caruntu I.D., Danciu M. (2015). Renal carcinogenesis—Insights into signaling pathways. Rom. J. Morphol. Embryol..

[7] Majid S., Saini S., Dahiya R. (2012). Wnt signaling pathways in urological cancers: Past decades and still growing. Mol. Cancer.

[8] Anastas J.N., Moon R.T. (2013). WNT signalling pathways as therapeutic targets in cancer. Nat. Rev. Cancer.

[9] Schambony A., Wedlich D. (2007). Wnt-5A/Ror2 regulate expression of XPAPC through an alternative noncanonical signaling pathway. Dev. Cell.

[10] Willert K., Jones K.A. (2006). Wnt signaling: Is the party in the nucleus?. Genes Dev..

[11] Aberle H., Bauer A., Stappert J., Kispert A., Kemler R. (1997). Beta-catenin is a target for the ubiquitin-proteasome pathway. EMBO J..

[12] Gordon M.D., Nusse R. (2006). Wnt signaling: Multiple pathways, multiple receptors, and multiple transcription factors. J. Biol. Chem..

[13] Stamos J.L., Weis W.I. (2013). The Beta-catenin destruction complex. Cold Spring Harb. Perspect. Biol..

[14] MacDonald B.T., Tamai K., He X. (2009). Wnt/Beta-catenin signaling: Components, mechanisms, and diseases. Dev. Cell..

[15] Gomez-Orte E., Saenz-Narciso B., Moreno S., Cabello J. (2013). Multiple functions of the noncanonical Wnt pathway. Trends Genet..

[16] Rao T.P., Kuhl M. (2010). An updated overview on Wnt signaling pathways: A prelude for more. Circ. Res..

[17] Huang L., Xiao A., Choi S.Y., Kan Q., Zhou W., Chacon-Heszele M.F., Ryu Y.K., McKenna S., Zuo X., Kuruvilla R. (2014). Wnt5a is necessary for normal kidney development in zebrafish and mice. Nephron Exp. Nephrol..

[18] Nishita M., Qiao S., Miyamoto M., Okinaka Y., Yamada M., Hashimoto R., Iijima K., Otani H., Hartmann C., Nishinakamura R. (2014). Role of Wnt5a-Ror2 signaling in morphogenesis of the metanephric mesenchyme during ureteric budding. Mol. Cell. Biol..

[19] Mesar I., Kes P., Jukic N.B. (2012). A role of Wnt in kidney development and function. Acta Med. Croatica.

[20] Vainio S.J. (2003). Nephrogenesis regulated by Wnt signaling. J. Nephrol..

[21] Caprioli A., Villasenor A., Wylie L.A., Braitsch C., Marty-Santos L., Barry D., Karner C.M., Fu S., Meadows S.M., Carroll T.J. (2015). Wnt4 is essential to normal mammalian lung development. Dev. Biol..

[22] Hernandez Gifford J.A. (2015). The role of Wnt signaling in adult ovarian folliculogenesis. Reproduction.

[23] Naillat F., Yan W., Karjalainen R., Liakhovitskaia A., Samoylenko A., Xu Q., Sun Z., Shen B., Medvinsky A., Quaggin S. (2015). Identification of the genes regulated by Wnt-4, a critical signal for commitment of the ovary. Exp. Cell Res..

[24] Prunskaite-Hyyrylainen R., Skovorodkin I., Xu Q., Miinalainen I., Shan J., Vainio S.J. (2016). Wnt4 coordinates directional cell migration and extension of the mullerian duct essential for ontogenesis of the female reproductive tract. Hum. Mol. Genet..

[25] Prunskaite-Hyyrylainen R., Shan J., Railo A., Heinonen K.M., Miinalainen I., Yan W., Shen B., Perreault C., Vainio S.J. (2014). Wnt4, a pleiotropic signal for controlling cell polarity, basement membrane integrity, and antimullerian hormone expression during oocyte maturation in the female follicle. FASEB J..

[26] Balic A., Thesleff I. (2015). Tissue interactions regulating tooth development and renewal. Curr. Top. Dev. Biol..

[27] Fujimori S., Novak H., Weissenbock M., Jussila M., Goncalves A., Zeller R., Galloway J., Thesleff I., Hartmann C. (2010). Wnt/Beta-catenin signaling in the dental mesenchyme regulates incisor development by regulating BMP4. Dev. Biol..

[28] Jussila M., Thesleff I. (2012). Signaling networks regulating tooth organogenesis and regeneration, and the specification of dental mesenchymal and epithelial cell lineages. Cold Spring Harb. Perspect. Biol..

[29] Yao D., Dai C., Peng S. (2011). Mechanism of the mesenchymal-epithelial transition and its relationship with metastatic tumor formation. Mol. Cancer Res..

[30] Yang Z., Balic A., Michon F., Juuri E., Thesleff I. (2015). Mesenchymal Wnt/Beta-catenin signaling controls epithelial stem cell homeostasis in teeth by inhibiting the antiapoptotic effect of FGF10. Stem Cells.

[31] Afelik S., Pool B., Schmerr M., Penton C., Jensen J. (2015). Wnt7b is required for epithelial progenitor growth and operates during epithelial-to-mesenchymal signaling in pancreatic development. Dev. Biol..

[32] Zhang H., Zhou W.C., Li X., Meng W.B., Zhang L., Zhu X.L., Zhu K.X., Bai Z.T., Yan J., Liu T. (2014). 5-Azacytidine suppresses the proliferation of pancreatic cancer cells by inhibiting the Wnt/Beta-catenin signaling pathway. Genet. Mol. Res..

[33] Gurskaya O.Y., Dobryakova Y.V., Markevich V.A. (2015). A role of the Wnt signaling in the regulation of brain function. Zh. Vyssh. Nerv. Deiat. Im. I. P. Pavlova.

[34] Boras-Granic K., Hamel P.A. (2013). Wnt-signalling in the embryonic mammary gland. J. Mammary Gland Biol. Neoplasia.

[35] Lim X., Nusse R. (2013). Wnt signaling in skin development, homeostasis, and disease. Cold Spring Harb. Perspect. Biol..

[36] Koles K., Budnik V. (2012). Wnt signaling in neuromuscular junction development. Cold Spring Harb. Perspect. Biol..

[37] Clevers H., Nusse R. (2012). Wnt/Beta-catenin signaling and disease. Cell.

[38] Logan C.Y., Nusse R. (2004). The Wnt signaling pathway in development and disease. Annu. Rev. Cell Dev. Biol..

[39] Shan J., Jokela T., Peltoketo H., Vainio S. (2009). Generation of an allele to inactivate Wnt4 gene function conditionally in the mouse. Genesis.

[40] Pietila I., Prunskaite-Hyyrylainen R., Kaisto S., Tika E., van Eerde A.M., Salo A.M., Garma L., Miinalainen I., Feitz W.F., Bongers E.M. (2016). Wnt5a deficiency leads to anomalies in ureteric tree development, tubular epithelial cell organization and basement membrane integrity pointing to a role in kidney collecting duct patterning. PLoS ONE.

[41] Majumdar A., Vainio S., Kispert A., McMahon J., McMahon A.P. (2003). Wnt11 and Ret/Gdnf pathways cooperate in regulating ureteric branching during metanephric kidney development. Development.

[42] Nwabo Kamdje A.H., Seke Etet P.F., Vecchio L., Muller J.M., Krampera M., Lukong K.E. (2014). Signaling pathways in breast cancer: Therapeutic targeting of the microenvironment. Cell. Signal..

[43] Guillen-Ahlers H. (2008). Wnt signaling in renal cancer. Curr. Drug Targets.

[44] Duchartre Y., Kim Y.M., Kahn M. (2016). The Wnt signaling pathway in cancer. Crit. Rev. Oncol. Hematol..

[45] Kruck S., Eyrich C., Scharpf M., Sievert K.D., Fend F., Stenzl A., Bedke J. (2013). Impact of an Altered Wnt1/Beta-catenin expression on clinicopathology and prognosis in clear cell renal cell carcinoma. Int. J. Mol. Sci..

[46] Hsu R.J., Ho J.Y., Cha T.L., Yu D.S., Wu C.L., Huang W.P., Chu P., Chen Y.H., Chen J.T., Yu C.P. (2012). WNT10A plays an oncogenic role in renal cell carcinoma by activating WNT/Beta-catenin pathway. PLoS ONE.

[47] Kondratov A.G., Kvasha S.M., Stoliar L.A., Romanenko A.M., Zgonnyk Y.M., Gordiyuk V.V., Kashuba E.V., Rynditch A.V., Zabarovsky E.R., Kashuba V.I. (2012). Alterations of the Wnt7a gene in clear cell renal cell carcinomas. PLoS ONE.

[48] Tamimi Y., Ekuere U., Laughton N., Grundy P. (2008). WNT5A is regulated by PAX2 and may be involved in blastemal predominant wilms tumorigenesis. Neoplasia.

[49] Shimizu H., Julius M.A., Giarre M., Zheng Z., Brown A.M., Kitajewski J. (1997). Transformation by Wnt family proteins correlates with regulation of Beta-catenin. Cell Growth Differ..

[50] Yuzugullu H., Benhaj K., Ozturk N., Senturk S., Celik E., Toylu A., Tasdemir N., Yilmaz M., Erdal E., Akcali K.C. (2009). Canonical Wnt signaling is antagonized by noncanonical Wnt5a in hepatocellular carcinoma cells. Mol. Cancer.

[51] Mizushima T., Nakagawa H., Kamberov Y.G., Wilder E.L., Klein P.S., Rustgi A.K. (2002). Wnt-1 but not epidermal growth factor induces Beta-catenin/T-Cell factor-dependent transcription in esophageal cancer cells. Cancer Res..

[52] Siar C.H., Nagatsuka H., Han P.P., Buery R.R., Tsujigiwa H., Nakano K., Ng K.H., Kawakami T. (2012). Differential expression of canonical and non-canonical Wnt ligands in ameloblastoma. J. Oral Pathol. Med..

[53] Janssens N., Andries L., Janicot M., Perera T., Bakker A. (2004). Alteration of Frizzled expression in renal cell carcinoma. Tumour Biol..

[54] Long Q.Z., Du Y.F., Liu X.G., Li X., He D.L. (2015). miR-124 represses FZD5 to attenuate P-glycoprotein-mediated chemo-resistance in renal cell carcinoma. Tumour Biol..

[55] Xu R., Zeng S., Xie W., Sun C., Chen Y.L., Chen M.J., Zhang L. (2016). The expression and function of Frizzled-7 in human renal cell carcinoma. Clin. Transl. Oncol..

[56] Bilim V., Kawasaki T., Katagiri A., Wakatsuki S., Takahashi K., Tomita Y. (2000). Altered expression of Beta-catenin in renal cell cancer and transitional cell cancer with the absence of Beta-catenin gene mutations. Clin. Cancer Res..

[57] Ueda M., Gemmill R.M., West J., Winn R., Sugita M., Tanaka N., Ueki M., Drabkin H.A. (2001). Mutations of the Beta- and Gamma-catenin genes are uncommon in human lung, breast, kidney, cervical and ovarian carcinomas. Br. J. Cancer.

[58] Kim Y.S., Kang Y.K., Kim J.B., Han S.A., Kim K.I., Paik S.R. (2000). Beta-catenin expression and mutational analysis in renal cell carcinomas. Pathol. Int..

[59] Arai E., Sakamoto H., Ichikawa H., Totsuka H., Chiku S., Gotoh M., Mori T., Nakatani T., Ohnami S., Nakagawa T. (2014). Multilayer-omics analysis of renal cell carcinoma, including the whole exome, methylome and transcriptome. Int. J. Cancer.

[60] Chen S., Guttridge D.C., You Z., Zhang Z., Fribley A., Mayo M.W., Kitajewski J., Wang C.Y. (2001). Wnt-1 signaling inhibits apoptosis by activating Beta-catenin/T cell factor-mediated transcription. J. Cell Biol..

[61] Shiina H., Igawa M., Breault J., Ribeiro-Filho L., Pookot D., Urakami S., Terashima M., Deguchi M., Yamanaka M., Shirai M. (2003). The human T-cell factor-4 gene splicing isoforms, Wnt signal pathway, and apoptosis in renal cell carcinoma. Clin. Cancer Res..

[62] Saini S., Majid S., Dahiya R. (2011). The complex roles of Wnt antagonists in RCC. Nat. Rev. Urol..

[63] Hirata H., Hinoda Y., Majid S., Chen Y., Zaman M.S., Ueno K., Nakajima K., Tabatabai Z.L., Ishii N., Dahiya R. (2011). DICKKOPF-4 activates the noncanonical c-Jun-NH2 kinase signaling pathway while inhibiting the Wnt-canonical pathway in human renal cell carcinoma. Cancer.

[64] Kawakami K., Yamamura S., Hirata H., Ueno K., Saini S., Majid S., Tanaka Y., Kawamoto K., Enokida H., Nakagawa M. (2011). Secreted frizzled-related protein-5 is epigenetically downregulated and functions as a tumor suppressor in kidney cancer. Int. J. Cancer.

[65] Hirata H., Hinoda Y., Nakajima K., Kikuno N., Yamamura S., Kawakami K., Suehiro Y., Tabatabai Z.L., Ishii N., Dahiya R. (2009). Wnt antagonist gene polymorphisms and renal cancer. Cancer.

[66] Kawakami K., Hirata H., Yamamura S., Kikuno N., Saini S., Majid S., Tanaka Y., Kawamoto K., Enokida H., Nakagawa M. (2009). Functional significance of Wnt inhibitory factor-1 gene in kidney cancer. Cancer Res..

[67] Gumz M.L., Zou H., Kreinest P.A., Childs A.C., Belmonte L.S., LeGrand S.N., Wu K.J., Luxon B.A., Sinha M., Parker A.S. (2007). Secreted Frizzled-related protein 1 loss contributes to tumor phenotype of clear cell renal cell carcinoma. Clin. Cancer Res..

[68] Urakami S., Shiina H., Enokida H., Kawakami T., Tokizane T., Ogishima T., Tanaka Y., Li L.C., Ribeiro-Filho L.A., Terashima M. (2006). Epigenetic inactivation of Wnt inhibitory factor-1 plays an important role in bladder cancer through aberrant canonical Wnt/Beta-catenin signaling pathway. Clin. Cancer Res..

[69] Kawamoto K., Hirata H., Kikuno N., Tanaka Y., Nakagawa M., Dahiya R. (2008). DNA methylation and histone modifications cause silencing of Wnt antagonist gene in human renal cell carcinoma cell lines. Int. J. Cancer.

[70] Konac E., Varol N., Yilmaz A., Menevse S., Sozen S. (2013). DNA methyltransferase inhibitor-mediated apoptosis in the Wnt/Beta-catenin signal pathway in a renal cell carcinoma cell line. Exp. Biol. Med..

[71] Yamamura S., Kawakami K., Hirata H., Ueno K., Saini S., Majid S., Dahiya R. (2010). Oncogenic functions of secreted Frizzled-related protein 2 in human renal cancer. Mol. Cancer Ther..

[72] Esteve P., Bovolenta P. (2010). The advantages and disadvantages of SFRP1 and SFRP2 expression in pathological events. Tohoku J. Exp. Med..

[73] Guo C.C., Zhang X.L., Yang B., Geng J., Peng B., Zheng J.H. (2014). Decreased expression of DKK1 and DKK3 in human clear cell renal cell carcinoma. Mol. Med. Rep..

[74] Blish K.R., Clausen K.A., Hawkins G.A., Garvin A.J., Willingham M.C., Turner J.C., Torti F.M., Torti S.V. (2010). Loss of heterozygosity and SOSTDC1 in adult and pediatric renal tumors. J. Exp. Clin. Cancer Res..

[75] Ueno K., Hirata H., Majid S., Tabatabai Z.L., Hinoda Y., Dahiya R. (2011). IGFBP-4 activates the Wnt/Beta-catenin signaling pathway and induces M-CAM expression in human renal cell carcinoma. Int. J. Cancer.

[76] Furge K.A., Chen J., Koeman J., Swiatek P., Dykema K., Lucin K., Kahnoski R., Yang X.J., Teh B.T. (2007). Detection of DNA copy number changes and oncogenic signaling abnormalities from gene expression data reveals MYC activation in high-grade papillary renal cell carcinoma. Cancer Res..

[77] Peruzzi B., Athauda G., Bottaro D.P. (2006). The Von Hippel-Lindau tumor suppressor gene product represses oncogenic Beta-catenin signaling in renal carcinoma cells. Proc. Natl. Acad. Sci. USA.

[78] Gossage L., Eisen T., Maher E.R. (2015). VHL, the story of a tumour suppressor gene. Nat. Rev. Cancer.

[79] Schödel J., Grampp S., Maher E.R., Moch H., Ratcliffe P.J., Russo P., Mole D.R. (2016). Hypoxia, hypoxia-inducible transcription factors, and renal cancer. Eur. Urol..

[80] Wright T.M., Brannon A.R., Gordan J.D., Mikels A.J., Mitchell C., Chen S., Espinosa I., van de Rijn M., Pruthi R., Wallen E. (2009). ROR2, a developmentally regulated kinase, promotes tumor growth potential in renal cell carcinoma. Oncogene.

[81] Rasmussen N.R., Debebe Z., Wright T.M., Brooks S.A., Sendor A.B., Brannon A.R., Hakimi A.A., Hsieh J.J., Choueiri T.K., Tamboli P. (2014). Expression of ROR2 mediates invasive phenotypes in renal cell carcinoma. PLoS ONE.

[82] Ueno K., Hirata H., Majid S., Chen Y., Zaman M.S., Tabatabai Z.L., Hinoda Y., Dahiya R. (2011). Wnt antagonist DICKKOPF-3 (Dkk-3) induces apoptosis in human renal cell carcinoma. Mol. Carcinog..

[83] Thun M.J., DeLancey J.O., Center M.M., Jemal A., Ward E.M. (2010). The global burden of cancer: Priorities for prevention. Carcinogenesis.

[84] Keshet E., Ben-Sasson S.A. (1999). Anticancer drug targets: Approaching angiogenesis. J. Clin. Invest..

[85] Shapiro G.I., Harper J.W. (1999). Anticancer drug targets: Cell cycle and checkpoint control. J. Clin. Invest..

[86] Coppin C., Le L., Porzsolt F., Wilt T. (2008). Targeted therapy for advanced renal cell carcinoma. Cochrane Database Syst. Rev..

[87] Zhang L., Bhasin M., Schor-Bardach R., Wang X., Collins M.P., Panka D., Putheti P., Signoretti S., Alsop D.C., Libermann T. (2011). Resistance of renal cell carcinoma to sorafenib is mediated by potentially reversible gene expression. PLoS ONE.

[88] Ho J.Y., Hsu R.J., Wu C.L., Chang W.L., Cha T.L., Yu D.S., Yu C.P. (2013). Ovatodiolide targets Beta-catenin signaling in suppressing tumorigenesis and overcoming drug resistance in renal cell carcinoma. Evid. Based. Complement. Alternat. Med..

[89] Sasamura H., Takahashi A., Yuan J., Kitamura H., Masumori N., Miyao N., Itoh N., Tsukamoto T. (2004). Antiproliferative and antiangiogenic activities of genistein in human renal cell carcinoma. Urology.

[90] Hirata H., Ueno K., Nakajima K., Tabatabai Z.L., Hinoda Y., Ishii N., Dahiya R. (2013). Genistein Downregulates Onco-miR-1260b and inhibits Wnt-signalling in renal cancer cells. Br. J. Cancer.

[91] Hillman G.G., Wang Y., Che M., Raffoul J.J., Yudelev M., Kucuk O., Sarkar F.H. (2007). Progression of renal cell carcinoma is inhibited by genistein and radiation in an orthotopic model. BMC Cancer.

[92] Gagan J., Van Allen E.M. (2015). Next-generation sequencing to guide cancer therapy. Genome Med..

[93] Morrison P.J., Donnelly D.E., Atkinson A.B., Maxwell A.P. (2010). Advances in the genetics of familial renal cancer. Oncologist.

[94] Toschi L., Janne P.A. (2008). Single-agent and combination therapeutic strategies to inhibit hepatocyte growth factor/MET signaling in cancer. Clin. Cancer Res..

[95] Koller C.M., Kim Y., Schmidt-Wolf I.G. (2013). Targeting renal cancer with a combination of Wnt inhibitors and a Bi-functional peptide. Anticancer Res..

[96] Von Schulz-Hausmann S.A., Schmeel L.C., Schmeel F.C., Schmidt-Wolf I.G. (2014). Targeting the Wnt/Beta-catenin pathway in renal cell carcinoma. Anticancer Res..

[97] Khan M.I., Czarnecka A.M., Helbrecht I., Bartnik E., Lian F., Szczylik C. (2015). Current approaches in identification and isolation of human renal cell carcinoma cancer stem cells. Stem Cell. Res. Ther..

[98] Takebe N., Miele L., Harris P.J., Jeong W., Bando H., Kahn M., Yang S.X., Ivy S.P. (2015). Targeting Notch, Hedgehog, and Wnt pathways in cancer stem cells: Clinical update. Nat. Rev. Clin. Oncol..

[99] Smith B.H., Gazda L.S., Conn B.L., Jain K., Asina S., Levine D.M., Parker T.S., Laramore M.A., Martis P.C., Vinerean H.V. (2011). Three-dimensional culture of mouse renal carcinoma cells in agarose macrobeads selects for a subpopulation of cells with cancer stem cell or cancer progenitor properties. Cancer Res..

[100] Blagodatski A., Poteryaev D., Katanaev V.L. (2014). Targeting the Wnt pathways for therapies. Mol. Cell. Ther..

[101] Madan B., Virshup D.M. (2015). Targeting Wnts at the source—New mechanisms, new biomarkers, new drugs. Mol. Cancer Ther..

[102] Kahn M. (2014). Can we safely target the Wnt pathway?. Nat. Rev. Drug Discov..

[103] Rak-Raszewska A., Hauser P.V., Vainio S. (2015). Organ *in vitro* culture: What have we learned about early kidney development?. Stem Cells Int..

[104] Unbekandt M., Davies J.A. (2010). Dissociation of embryonic kidneys followed by reaggregation allows the formation of renal tissues. Kidney Int..

[105] Junttila S., Saarela U., Halt K., Manninen A., Parssinen H., Lecca M.R., Brandli A.W., Sims-Lucas S., Skovorodkin I., Vainio S.J. (2015). Functional genetic targeting of embryonic kidney progenitor cells *ex vivo*. J. Am. Soc. Nephrol..

[106] Takasato M., Er P.X., Chiu H.S., Maier B., Baillie G.J., Ferguson C., Parton R.G., Wolvetang E.J., Roost M.S., Chuva de Sousa Lopes S.M. (2015). Kidney organoids from human iPS cells contain multiple lineages and model human nephrogenesis. Nature.

[107] Gross J.C., Chaudhary V., Bartscherer K., Boutros M. (2012). Active Wnt proteins are secreted on exosomes. Nat. Cell Biol..

[108] Raposo G., Stoorvogel W. (2013). Extracellular vesicles: Exosomes, microvesicles, and friends. J. Cell Biol..

[109] Yanez-Mo M., Siljander P.R., Andreu Z., Zavec A.B., Borras F.E., Buzas E.I., Buzas K., Casal E., Cappello F., Carvalho J. (2015). Biological properties of extracellular vesicles and their physiological functions. J. Extracell Vesicles.

[110] Krause M., Samoylenko A., Vainio S.J. (2015). Exosomes as renal inductive signals in health and disease, and their application as diagnostic markers and therapeutic agents. Front. Cell. Dev. Biol..

[111] Du T., Ju G., Wu S., Cheng Z., Cheng J., Zou X., Zhang G., Miao S., Liu G., Zhu Y. (2014). Microvesicles derived from human wharton’s Jelly mesenchymal stem cells promote human renal cancer cell growth and aggressiveness through induction of hepatocyte growth factor. PLoS ONE.

[112] Luga V., Zhang L., Viloria-Petit A.M., Ogunjimi A.A., Inanlou M.R., Chiu E., Buchanan M., Hosein A.N., Basik M., Wrana J.L. (2012). Exosomes mediate stromal mobilization of autocrine Wnt-PCP signaling in breast cancer cell migration. Cell.

